# Rhythm Analysis during Cardiopulmonary Resuscitation Using Convolutional Neural Networks

**DOI:** 10.3390/e22060595

**Published:** 2020-05-27

**Authors:** Iraia Isasi, Unai Irusta, Elisabete Aramendi, Trygve Eftestøl, Jo Kramer-Johansen, Lars Wik

**Affiliations:** 1 Department of Communications Engineering, University of the Basque Country UPV/EHU, 48013 Bilbao, Spain; unai.irusta@ehu.eus (U.I.); elisabete.aramendi@ehu.eus (E.A.); 2 Department of Electrical Engineering and Computer Science, University of Stavanger, 4036 Stavanger, Norway; trygve.eftestol@uis.no; 3 Norwegian National Advisory Unit on Prehospital Emergency Medicine (NAKOS), Oslo University Hospital and University of Oslo, 0424 Oslo, Norway; jo.kramer-johansen@medisin.uio.no (J.K.-J.); lars.wik@medisin.uio.no (L.W.)

**Keywords:** out-of-hospital cardiac arrest (OHCA), cardiopulmonary resuscitation (CPR), electrocardiogram (ECG), adaptive filter, deep learning, machine learning, convolutional neural network (CNN), random forest (RF) classifier

## Abstract

Chest compressions during cardiopulmonary resuscitation (CPR) induce artifacts in the ECG that may provoque inaccurate rhythm classification by the algorithm of the defibrillator. The objective of this study was to design an algorithm to produce reliable shock/no-shock decisions during CPR using convolutional neural networks (CNN). A total of 3319 ECG segments of 9 s extracted during chest compressions were used, whereof 586 were shockable and 2733 nonshockable. Chest compression artifacts were removed using a Recursive Least Squares (RLS) filter, and the filtered ECG was fed to a CNN classifier with three convolutional blocks and two fully connected layers for the shock/no-shock classification. A 5-fold cross validation architecture was adopted to train/test the algorithm, and the proccess was repeated 100 times to statistically characterize the performance. The proposed architecture was compared to the most accurate algorithms that include handcrafted ECG features and a random forest classifier (baseline model). The median (90% confidence interval) sensitivity, specificity, accuracy and balanced accuracy of the method were 95.8% (94.6–96.8), 96.1% (95.8–96.5), 96.1% (95.7–96.4) and 96.0% (95.5–96.5), respectively. The proposed algorithm outperformed the baseline model by 0.6-points in accuracy. This new approach shows the potential of deep learning methods to provide reliable diagnosis of the cardiac rhythm without interrupting chest compression therapy.

## 1. Introduction

Out of hospital cardiac arrest (OHCA) is one of the leading causes of death worldwide [1,2]. The two key life saving therapies are defibrillation (electric shock) when the cardiac rhythm is ventricular fibrillation (VF) or tachycardia (VT), and cardiopulmonary resuscitation (CPR) [3]. The defibrillator monitors the electrocardiogram (ECG), and includes a shock/no-shock algorithm that analyzes the patient’s ECG to detect VF/VT [4]. The American Heart Association (AHA) has established the minimum accuracy requirements for these algorithms [5]. Shockable rhythms should be detected with a minimum sensitivity (Se) of 90% to properly identify defibrillation treatment conditions. The specificity (Sp) for detection of nonshockable rhythms must be above 95% to avoid unnecessary shocks that may damage the myocardium or deteriorate the quality of CPR.

The mechanical activity of chest compressions during CPR induces artifacts in the ECG that impede a reliable shock/no-shock decision by the defibrillator [6]. Therefore, defibrillators prompt the rescuers to stop chest compressions for rhythm analysis every 2 minutes [7,8]. These hands off (or no flow) intervals lead to intermittent periods with no cerebral and myocardial blood flow that deteriorate the patient’s condition, and compromise survival [7,9,10,11]. Consequently, many biomedical engineering solutions have been proposed over the years to allow an AHA compliant shock/no-shock decision during CPR [12], but none of these solutions have yet a sufficient positive predictivity to be implemented in commercial defibrillators. These methods are based on adaptive filters to remove CPR artifacts. Adaptive filters are needed to address the time and frequency variability of the artifact and its spectral overlap with OHCA rhythms [13]. These filters use signals recorded by the defibrillator like compression depth (CD) or thoracic impedance (TI) to model the artifact [14,15]. Several adaptive approaches have been demonstrated including Wiener filters [16], Matching Pursuit Algorithms [17], Recursive Least Squares (RLS) [18], Least Mean Squares (LMS) [19], or Kalman filters [20,21]. Once the artifact is removed the ECG is analyzed using the shock/no-shock algorithms in defibrillators, or ad-hoc algorithms specially designed to analyze the filtered ECG [17,19,22]. The latter have shown the highest Se/Sp values by exploiting recent advances in ECG feature extraction and classical machine learning algorithms. ECG features are customarily computed in time, frequency or time-frequency domains [23,24,25,26]. These features have been efficiently combined using classical machine learning classification algorithms like support vector machines (SVM) or random forests (RF) [22,25,26].

Recently, deep learning approaches have proven to be superior to classical machine learning algorithms in many biomedical signal applications [27,28], including arrhythmia classification based on the ECG waveform [29,30,31,32,33]. Deep learning algorithms using convolutional neural networks (CNN) are end-to-end solutions in which the algorithm learns efficient internal representations of the data (features) and combines them to solve the classification task [34,35]. Deep learning algorithms have already been shown to outperform classical machine learning algorithms in some OHCA applications, such as detection of VF in artifact free ECG [30,36], or the detection of pulse [37]. However, deep learning has not been applied to design algorithms that give accurate shock/no-shock decisions during CPR.

The objective of this study was to design the first deep learning solution to discriminate shockable from nonshockable rhythms during CPR. The method comprises two stages, an adaptive RLS filter to remove CPR artifacts from the ECG followed by a CNN to classify the filtered ECG. The paper is organized as follows: the study dataset is detailed in Section 2, Section 3 describes the methodology including the CNN architecture and the evaluation procedure. The results are presented in Section 4, discussed in Section 5 and the main conclusions are presented in Section 6.

## 2. Materials

**Figure 1:**
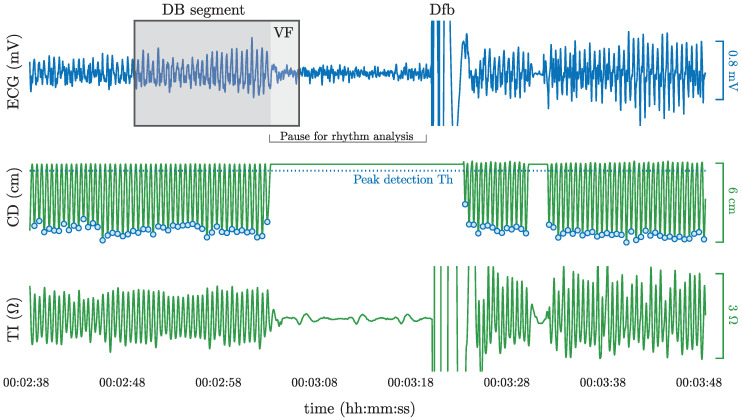
A 70 s interval from an OHCA episode showing the ECG, CD and TI signals. Activity shows CPR followed by a pause for rhythm analysis, the delivery of a defibrillation shock (Dfb) and immediate resumption of CPR. The interval highlighted in grey corresponds to a 15.5 s segment in the dataset. During the first 12.5 s of the segment chest compressions were delivered (see activity in TI and CD), and in the last 3 s there were no compressions and the ground truth rhythm (VF) for the whole segment could be annotated.

Data were extracted from a large prospective clinical trial designed to measure CPR quality during OHCA [38]. The study was conducted between March 2002 and September 2004 by the emergency services of London, Stockholm and Akershus (Norway). CPR was performed using prototype defibrillators based on HeartStart 4000 (Philips Medical Systems, Andover, MA, USA) together with a sternal CPR assist pad fitted with an accelerometer (ADXL202e, AnalogDevice, Norwood, Mass). The raw data for this study consisted of the ECG and TI signals acquired through the defibrillation pads and the CD signal derived from accelerometer data [16]. Defibrillator data was anonymized and converted to Matlab (MathWorks Inc, Natick, MA, USA) using a sampling rate of 250 Hz. The ECG had an amplitude resolution of 1.031 μV per least significant bit. A notch filter and a Hampel filter were used to remove powerline interferences and spiky artifacts from the ECG [37]. Finally, chest compressions instants ($t_{k}$) were automatically marked using a negative peak detector with a 1 cm threshold on the CD signal (see Figure 1, peak detection Th) [15].

The rhythms in the OHCA episodes were originally annotated by two experienced resuscitation researchers/practitioners, a biomedical engineer and an anesthesiologist [38]. For the purpose of this study the rhythm annotations were grouped into shockable and nonshockable. Shockable rhythms comprised lethal ventricular arrhythmia, predominantly VF but also pulseless VT. Non-shockable rhythms included asystole (AS), the absence of electrical activity, and organized rhythms (ORG), or rhythms with visible QRS complexes. The OHCA episodes had median (interquartile range, IQR) durations of 26 min (17–33). From these episodes 15.5 s segments were automatically extracted following these criteria: unique rhythm type in the segment and an interval of 12.5 s with ongoing compressions followed or preceded by a 3 s interval without compressions. The 12.5 s interval with ongoing compressions was used to develop the shock/no-shock decision algorithm, and the 3 s segment was used to confirm the original rhythm annotation in an artifact free ECG. All the data were visually revised (double blind process by authors UI and TE) to ensure compliance with the extraction criteria and the correctness of the rhyhm annotations. The annotated dataset contained 3319 segments from 272 OHCA patients, whereof 586 were shockable and 2733 (1192 AS and 1541 ORG) were nonshockable.

## 3. Methods

The shock/no-shock decision algorithms proposed in this study are composed of two stages. First, an adaptive RLS filter was used to remove chest compression artifacts from the ECG. Then shock/no-shock decision algorithms were designed to classify the filtered ECG using CNNs. In what follows $t=n\;\cdot \;T_{s}$, where $T_{s}$ = 4 ms is the sampling period ($f_{s}=$ 250 Hz), and *n* is the sample index.

### 3.1. CPR Artifact Suppressing Filter

**Figure 2:**
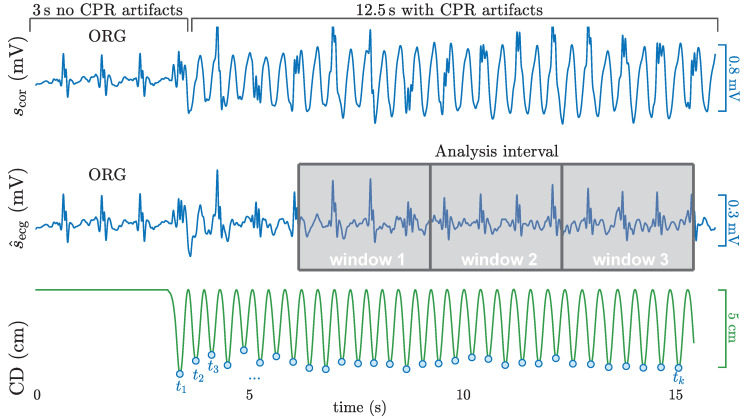
A 15.5 s segment from the study dataset corresponding to a patient in an organized rhythm is shown. In the initial 3 s interval without compressions three QRS complexes are visible, and the nonshockable rhythm annotation was confirmed. The following 12.5 s are corrupted by CPR artifacts (top panel) that conceal the underlying rhythm. The output of the adaptive filter, ${\hat{s}}_{\mathrm{ecg}}\left(n\right)$, reveals the underlying rhythm during chest compressions. CPR activity and the chest compression instants ($t_{k}$) can be observed in the CD signal (bottom).

CPR artifacts were suppressed using a state-of-the-art method [26,39] based on a RLS filter designed to remove periodic interferences [40]. The CPR artifact is modeled as a quasi-periodic interference using a Fourier series truncated to *N* terms (harmonics). The fundamental frequency of the artifact is that of the chest compressions [19], which is assumed constant during a chest compression, but variable from compression to compression. This means that for an interval between two successive compressions at time points, $t_{k-1}$ and $t_{k}$ (see Figure 2), the frequency can be expressed as

(1) $$f_{0}\left(n\right)=\frac{1}{t_{k}-t_{k-1}}\;t_{k-1}\leq nT_{s}<t_{k}$$

and the *N*-term Fourier series representation is then:

(2) $${\hat{s}}_{\mathrm{cpr}}\left(n\right)=A\left(n\right)\sum_{\ell =1}^{N}\left(\;a_{\ell }\left(n\right)\cos\left(\ell 2\pi f_{0}\left(n\right)T_{s}n\right)+b_{\ell }\left(n\right)\sin\left(\ell 2\pi f_{0}\left(n\right)T_{s}n\right)\;\right)$$

where A(n) is an amplitude envelope which differentiates intervals with (A=1) and without compressions (A=0), *N* is the number of harmonics in the Fourier series and $f_{0}\left(n\right)$ is the instantaneous chest compression frequency.

The RLS filter adaptively estimates the time-varying Fourier coefficients, $a_{\ell }\left(n\right)$ and $b_{\ell }\left(n\right)$, of the CPR artifact, ${\hat{s}}_{\mathrm{cpr}}\left(n\right)$, by minimizing in each iteration the error between the corrupted ECG, $s_{\mathrm{cor}}\left(n\right)$, and the estimated underlying ECG, ${\hat{s}}_{\mathrm{ecg}}\left(n\right)$, only around the spectral components of the CPR artifact, that is $f_{0}\left(n\right)$ and its harmonics. The underlying ECG is estimated assuming an additive noise model, so ${\hat{s}}_{\mathrm{ecg}}\left(n\right)=s_{\mathrm{cor}}\left(n\right)-{\hat{s}}_{\mathrm{cpr}}\left(n\right)$. A detailed description of the RLS filter equations is available in [39], and the values recommended there to suppress CPR artifacts were used in this study, that is, N=4 and a forgetting factor of λ=0.999 [39].

The shock/no-shock algorithms trained and evaluated in this study comprise algorithms based on CNNs (core methods of the paper), and a state of the art algorithm based on classical machine learning techniques used as a baseline model for comparison. In both cases, the algorithms were designed to analyze the filtered ECG in the interval from 3–12 s during compressions (see analysis interval in Figure 2). That is, the algorithms use 9 s of the filtered ECG for a decision, excluding the initial 3 s to avoid RLS filtering transients [39]. The analysis interval was further divided into three non-overlapping analysis windows of 3 s (see Figure 2) and the shock/no-shock decision was obtained as the majority vote. The combination of consecutive analysis windows is a typical design practice in shock/no-shock decision algorithms for defibrillators [41,42], because it increases the reliability of the diagnosis by avoiding the effects of transient lower quality signal intervals, rhythm changes or filtering miss-adjustments.

#### Algorithm Based on CNNs

**Figure 3:**
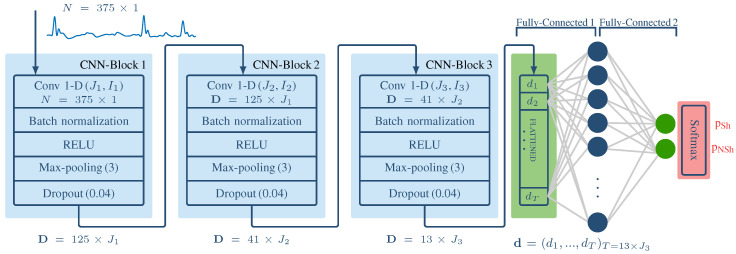
Architecture of the CNN-based shock/no-shock algorithm. It comprises two main stages: a CNN composed of three identical blocks and a classification stage based on two fully connected and a softmax layer.

Figure 3 shows the architecture of the shock/no-shock decision algorithms based on CNNs. First the 3 s window of the filtered ECG is downsampled to 125 Hz, resulting in a 1-D signal of *N* = 375 samples, ${\hat{s}}_{\mathrm{ecg}}\left(n\right)$. Then the CNN is composed of three convolutional blocks to extract the high level descriptors of the ECG, and two fully connected layers for classification. The *b*-th convolutional block consists of a convolutional layer with $J_{b}$ filters of width $I_{b}$, followed by a batch normalization layer, a rectified linear unit (ReLU), a max-pooling layer (K = 3) and a dropout layer.

### 3.2. Shock/No-Shock Decision Algorithms

Let us denote by $s_{b-1}\left(n,m\right)$ the output of block b−1 (input to block *b*), where *n* is the time index and *m* the filter index. In the first block the input is $s_{0}\left(n,1\right)={\hat{s}}_{\mathrm{ecg}}\left(n\right)$. The output of the Conv-1D layer at block *b* can be expressed as

(3) $$c_{b}\left(n,m\right)=f\left(b_{m}+\sum_{\ell =1}^{J_{b-1}}\sum_{i=1}^{I_{b}}\omega_{\ell ,i}^{m}\;s_{b-1}\left(n+i-1,\ell \right)\right)$$

where $\omega_{\ell ,i}^{m}$ are the network weights (convolutional coefficients), and f(x)=max(x,0) is the ReLU activation function that makes the network non-linear. The max-pooling layer selects the largest sample in blocks of *K* samples along the time index *n* to give the output of block *b*:

(4) $$s_{b}\left(n,m\right)=\max{\left\{c_{b}\left(k,m\right)\right\}}_{k=(n-1)\;\cdot \;K,\cdots ,n\;\cdot \;K}$$

Padding was applied before the convolutional and the max-pooling layers, so the only reduction of the dimensionality occurs at the max-pooling layers (K=3). This means that the dimensions of the outputs at blocks b=1,2,3 where $\left(125,\;J_{1}\right)$, $\left(41,\;J_{2}\right)$ and $\left(13,\;J_{3}\right)$, respectively and that the number of learnable parameters (ω,b) at block *b* where $J_{b}\times I_{b}+J_{b}$.

The dropout layer at the end of each block has a regularization effect, and is used only during training to avoid overfitting. It temporarily deactivates a randomdly selected proportion of the network’s tunable parameters, and has been shown to improve performance by providing noisy inputs to the fully connected layers that help avoid overfitting [43].

The classification stage takes as input the flattened $13\times J_{3}$ features and feeds them into two fully-connected layers. The first one is composed by 10 hidden units whereas the second one uses 2 neurons for the 2-class classification task. In the second fully-connected layer a softmax function is used to convert the output of the last two neurons into two values in the [0,1] range that can be interpreted as the likelihood that the 3 s window is shockable ($p_{\mathrm{Sh}}$) or nonshockable ($p_{\mathrm{NSh}}$).

The weights and biases of every layer were optimized using stochastic gradient descent with a momentum of 0.8. The initial learning rate was fixed to 0.02 and it was reduced by a factor of 0.8 at every epoch. The training data were fed into the CNN in batches of 256, and 20 epochs were used to train the networks [44]. During training data was augmented by splitting each 9 s training segment into overlapping 3 s windows with a linearly spaced start between 0 s and 6 s of the segment. To address class imbalance the augmented number of windows per segment during training was fixed to 100 for shockable and 40 for nonshockable rhythms, respectively. The binary cross entropy was used as loss function during network optimization (training):

(5) $$L=\sum_{i}y_{i}\ln\left(p_{Sh_{i}}\right)+\left(1-y_{i}\right)\ln\left(1-p_{Sh_{i}}\right)$$

where $y_{i}=\left\{0:\mathrm{NSh},1:\mathrm{Sh}\right\}$ corresponds to the rhythm label of 3 s training window *i*.

#### Classical Machine Learning Shock/no-Shock Decision Algorithm for Baseline Comparison

The baseline machine learning shock/no-shock algorithm is a state of the art solution described in [25]. In short, the algorithm is based on multiresolution ECG analysis using the Stationary Wavelet Transform (SWT) for feature extraction, followed by a random forest (RF) classifier. The SWT decomposes the 3 s window into 7 sub-bands, and the denoised ECG is reconstructed using detail coefficients $d_{3}$ to $d_{7}$, i.e., an analysis band of 0.98–31.25 Hz. The daubechies mother wavelet was used for the analysis as recommended in [26]. The selection of the mother wavelet was not a critical for this problem as shown in [26]. The denoised ECG, $s_{\mathrm{den}}\left(n\right)$, and the detail coefficients $d_{3}$-$d_{7}$ were used to obtain twenty five ECG features, selected using recursive feature elimination from a set of over 200 features (consult [25] for the details). The most relevant features were classical VF detection features like VFleak or $x_{4}$ [22,45] computed from $s_{\mathrm{den}}$, and a rich set of features obtained from the detail coefficients ${\left\{d_{i}\right\}}_{i=3,\cdots ,7}$, such as: sample entropy ($\mathrm{SampEn}(d_{i})$), the mean and standard deviation of the absolute value of the signal ($\bar{|d_{i}|}$, $\sigma (|d_{i}|)$) and its slope ($\bar{|\Delta d_{i}|}$, $\sigma (|\Delta d_{i}|)$), and the Hjorth mobility ($\mathrm{Hmb}(d_{i})$) and complexity ($\mathrm{Hmc}(d_{i})$) indices [46]. A detailed description of the algorithm is found in [25], with a detailed bibliography for the computation of the features.

The parameters of the RF classifier were fixed to those recommended in [25], that is *B* = 500 trees, 5 predictors per split (standard in RF), and the minimum observations per leaf to 3 (to avoid growing excessively deep or overfit trees). To avoid class imbalance uniform priors were assigned and a cost function was introduced to penalize false shock classifications with a factor of 2.5 (similar to the shock/no-shock augmentation factor used in the CNN).

### 3.3. Evaluation

All the classification algorithms were trained/tested using 5-fold cross validation (CV). Folds were partitioned patient-wise to avoid training/test data leakage, and in a quasi-stratified way by ensuring that the shock/no-shock prevalences in all folds were at least 80% those of the whole dataset. The performance of the method was evaluated using the standard metrics for binary classification problems, taking the shockable class as positive and the nonshockable class as negative. For a 2×2 confusion matrix with true positives (TP), true negatives (TN), false positives (FP) and false negatives FN) the performance metrics were

(6) $$\begin{array}{cc} \mathrm{Se}=\frac{\mathrm{TP}}{\mathrm{TP}+\mathrm{FN}} & \;\mathrm{PPV}=\frac{\mathrm{TP}}{\mathrm{TP}+\mathrm{FP}} \end{array}$$

(7) $$\begin{array}{ccc} \mathrm{Sp}=\frac{\mathrm{TN}}{\mathrm{TN}+\mathrm{FP}} & \; & \mathrm{NPV}=\frac{\mathrm{TN}}{\mathrm{TN}+\mathrm{FN}} \end{array}$$

(8) $$\begin{array}{cc} \mathrm{Acc}=\frac{\mathrm{TP}+\mathrm{TN}}{\mathrm{TP}+\mathrm{FN}+\mathrm{TN}+\mathrm{FP}} & \;\mathrm{BAC}=\frac{1}{2}\left(\mathrm{Se}+\mathrm{Sp}\right) \end{array}$$

The Balanced Accuracy (BAC) was used as target performance metric to ensure both shockable and nonshockable rhythms were accurately identified (as recommended by the AHA) despite the large class imbalance in the data.

## 4. Results

### 4.1. Parameters of the CNN Architecture

**Figure 4:**
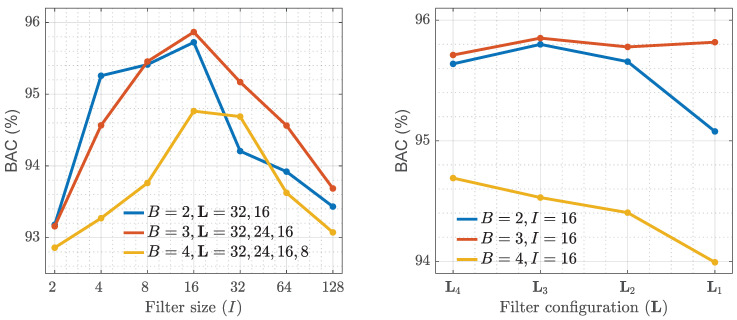
Performance of the CNN architecture for the configurable parameters of the network: the number of blocks (*B*), the filter size (*I*), and the filter configuration (L). The left panel shows the effect of the filter size for networks with $L_{4}=\left(32,24,16,8\right)$ filters. The right panel shows the effect of the filter configurations from dense ($L_{4}$) to sparse ($L_{1}$) for I=16.

The effect of changing the main parameters of the CNN architecture was first studied taking the BAC as target performance metric (see Figure 4). Three parameters were studied: the number of blocks (*B*), the size of the filters (*I*), and the number of filters ($L=(J_{1},\ldots J_{B})$). Four filter configurations were studied with decreasing number of filters (from dense to sparse): $L_{4}$ = (40, 30, 20, 10), $L_{3}$ = (32, 24, 16, 8), $L_{2}$ = (24, 18, 12, 6) and $L_{1}$ = (16, 12, 8, 4). The numbers in parentheses indicate the amount of filters from block 1 to block 4, so for arquitectures with 3 blocks and 2 blocks and $L_{2}$ the number of filters would be (24,18,12) and (24,12), respectively.

The results of the analysis are shown in Figure 4, with the median BAC computed over the 5-fold CV partitions. The best classification results were obtained for 3 blocks. Adding a fourth block increases the complexity (number of trainable parameters) and slightly decreases the performance. Using only 2 blocks resulted in a large decrease in performance (over 1-point in BAC), or an overly simplistic model. The best results for a CNN with 3 blocks were obtained with a filter width of I=16, and a filter configuration of L=(32,24,16). This was the CNN configuration adopted for the rest of the analyses.

### 4.2. Comparison with the Baseline Machine Learning Model

The shock/no-shock decision algorithms using CNNs and the classical machine learning model were compared. Table 1 shows the results for all the performance metrics. The accuracies were compared using McNemar’s test in all 5-fold CV partitions, and the results were considered significant at the 95% level. The CNN model was significantly more accurate (median *p* < 0.05) than the baseline model. As shown in Table 1, the CNN model designed for 9 s improves the best baseline models in 0.6-points in BAC and Acc, and in both cases the algorithms presented balanced Se/Sp values because they were trained to avoid class imbalance. The predictivity is higher for the CNN solution, but the differences are only large for shockable rhythms (PPV) because shockable rhythms have a much lower prevalence in the dataset (1 to 5). The table shows the results for the 3 s windows (where CNN outperforms the baseline model), but also for the combination of three consecutive analyses (9 s). For short windows the algorithms do not meet the minimum 95% value recommended by the AHA for artifact free ECG, but combining diagnoses with a majority vote criterion considerably improves performance and brings both the CNN solution and the baseline algorithm above AHA specifications. The table also shows the shock/no-shock decision performance when the two subgroups of nonshockable rhythms were evaluated separately, AS and ORG rhythms. The results show that no-shock decisions were more inaccurate when the underlying rhythm was asystole. For 9 s segments the CNN architecture yielded results slightly above the AHA’s 95% Sp goal for AS, but the baseline model was marginally below.

### 4.3. Effect of the ECG Corruption Level on Classification

CPR artifacts during chest compressions present very different noise levels in the ECG depending on variables like the position of the hands relative to the pads and cables, pad placement, or environmental conditions [47,48]. These variables are difficult to control in a pre-hospital setting, but it is important to know what the observed corruption levels are, and how these corruption levels affect the shock/no-shock decisions. To estimate the signal-to-noise ratio (SNR) the underlying ECG was assumed to be stationary over the 15 s segments, and thus the power of the clean signal ($P_{\mathrm{ecg}}$) was estimated in the 3 s interval without artifacts used to confirm the rhythm annotations. Then, CPR artifact estimated by the RLS filter was used to compute the power of the noise ($P_{\mathrm{cpr}}$), and to obtain the SNR as:

(9) $$\mathrm{SNR}=10\;\cdot \;\log_{10}\left(\frac{P_{\mathrm{ecg}}}{P_{\mathrm{cpr}}}\right)\;\left(\mathrm{dB}\right)$$

**Figure 5:**
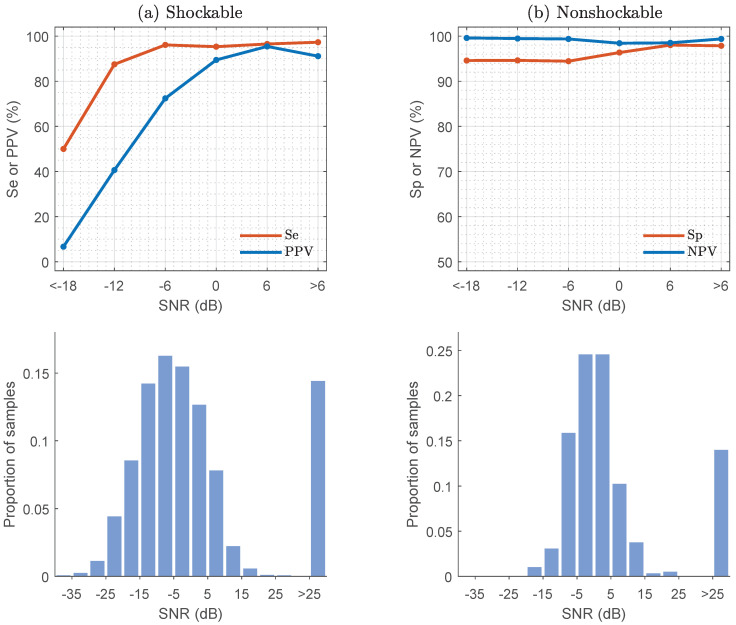
Median values of the performance metrics for shockable and nonshockable rhythms as a function of the SNR. The SNR levels were divided into 6dB bins for the analysis from high (<−18dB) to low (>6dB) corruption levels. The lower panels show the SNR distributions for shockable (**a**) and nonshockable rhythms (**b**).

The noise levels were divided into bins from very large corruption levels (SNR<−18dB) to very low corruption levels (SNR>6dB). The distributions of noise levels and the classification results for the different noise conditions are shown in Figure 5 for shockable (a) and nonshockable (b) rhythms. As expected the classification results improve as noise conditions improve, but noise affects the classification of shockable and nonshockable rhythms very differently. Nonshockable rhythms are detected with high specificity even in very noisy conditions, and the confidence in a nonshockable diagnosis (NPV) is high because the prevalence of nonshockable rhythms is 5/1 that of shockable rhythms. The sensitivity for shockable rhythms improves considerably as noise conditions improve, and was above the 90% value recommended by the AHA for SNR>−10dB. However, the confidence on a shock diagnosis (PPV) is good only for SNR>−6dB because of the lower prevalence of shockable rhythms. The SNR was significantly higher for nonshockable than for shockable rhythms (*p* < 0.001, Mann-Whitney U test), and in approximately 15% of shockable and nonshockable cases the noise level was negligible (SNR>25dB, see Figure 5). Although noise levels were lower in nonshockable rhythms, a high specificity was obtained regardless of the noise conditions. Even for the very noisy segments (SNR<−12dB) the specificity was above 94%.

### 4.4. Feature Extraction Using CNNs

**Figure 6:**
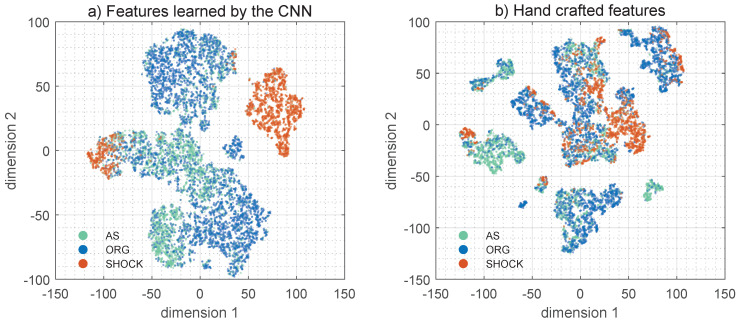
2D map representation of the separability of the classes for the features learned by the CNN (**a**) and the handcrafted features (**b**). These maps were obtained using the t-SNE algorithm.

For these experiments the 10 features at the output of the first fully connected layer were used as the features learned by the algorithm, these features will be named ${\left\{f_{i}\right\}}_{i=1,\cdots ,10}$. To evaluate feature extraction two experiments were conducted [36], and the results were compared to those obtained using the multiresolution features based on the SWT in the baseline model [25]. First, a dimensionality reduction experiment was conducted by projecting the feature space into a 2-D space using the t-distributed stochastic neighbor embedding (t-SNE) algorithm [49]. The results were visually assessed, and are shown in Figure 6 for the $f_{i}$ features and the handcrafted multiresolution features. The classes are shown in colors and the nonshockable rhythms are further divided into AS and ORG. As shown in the figure the CNN features produce better defined clusters than the handcrafted features in the 2D space. To numerically evaluate how the classes were clustered the Davies-Boudin index (DBi) was computed to measure the separability of the clusters [50]. The experiment was repeated on 500 bootstrap replicas and the mean (standard deviation) BDi for the CNN and the handcrafted features were 2.28 (0.06) and 4.95 (0.17), respectively (*p* < 0.05, for the paired t-test) [51]. That is, the features learned by the CNN architecture resulted in a more efficient clustering of the classes, and thus to a better separability.

Second, the discriminating power of each feature was computed using the area under the receiver characteristics curve (AUC). The results were obtained over 500 bootstrap replicas to statistically characterize the AUCs and compare the AUC distributions for each feature (paired t-test). The results are shown in Table 2, which shows that the four top most discriminating features ($f_{6}$, $f_{10}$, $f_{1}$ and $f_{5}$) had significantly higher AUCs (*p* < 0.05) than any of the handcrafted features. These results confirm the ability of the CNN to extract high quality discriminating features hidden in the signals.

### 4.5. Mixed Architectures

To further improve the BAC and accuracy of the CNN model three mixed architectures were also explored. First, the architecture of Figure 3 in which the softmax layer was replaced by a RF classifier to combine the best feature extraction (CNN) and classification (RF) of the the algorithms in Table 1, this solution was named CNN+RF. Second, a RF classifier fed with 25 handcrafted features and the 10 $f_{i}$ features was tested to see if handcrafted features added information to the CNN features, this was named All-Features. Finally, a basic stacking solution [52] in which the outputs of the CNN+RF (based on $f_{i}$) and the baseline model (handcrafted features) were used to form a majority vote (6 analyses, two per window), this solution was called Stacked. The results for 9 s segments are shown in Table 3, which shows that by using more elaborate solutions the BAC and Acc could be further improved in 0.4 and 0.5-points, respectively, either using all features or stacking the classifiers.

### 4.6. Analysis of Classification Errors

**Figure 7:**
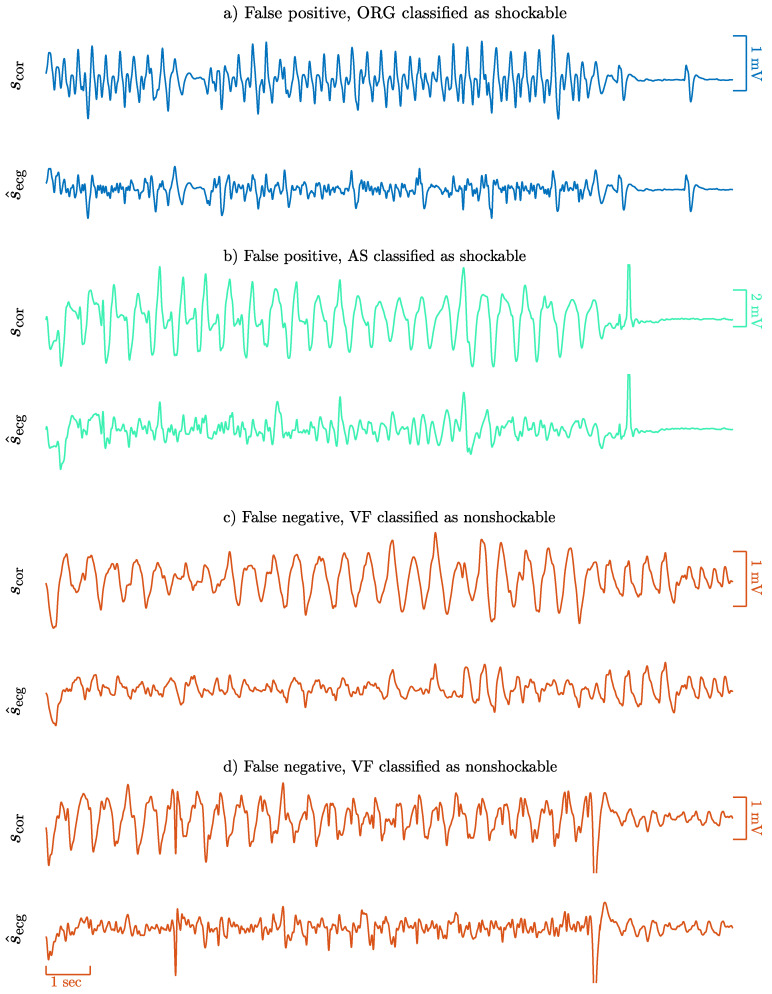
Examples of classification errors. The false positive examples (**a**,**b**) correspond to nonshockable rhythms classified as shockable (ORG panel a and AS panel b). The false negative examples (**c**,**d**) are shockable rhythms classified as nonshockable, and are shown in orange.

To conclude the analyses, the classification errors for the CNN based algorithm were identified. Some typical patterns leading to errors are shown in Figure 7. Most of the false positives are caused by the inability of the RLS filter to properly remove artifacts, leading to very disorganized filtering residuals that resemble a VF. Most false negatives occur at low SNR levels with compression rates around 100 min^−1^. In these cases the filtered ECG still shows an organized activity locked to the compression frequency, incompatible with fast ventricular arrhythmia and thus classified as non-shockable. Interestingly, these errors can be related to the clustering analysis of Section 4.4. Most errors cluster around borderline AS/VF rhythms which appear in the center-left region of the 2D t-SNE map (Figure 6), and ORG/VF rhythms in a much lower proportion in the top-center.

## 5. Discussion

This is, to the best of our knowledge, the first study that uses deep neural network models to discriminate between shockable and nonshockable rhythms during CPR. This algorithm consists of an adaptive RLS filter to remove CPR artifacts followed by a CNN to classify the filtered ECG. The algorithm designed for 9 s improves the performance of the classical machine learning algorithms by 0.6 points in BAC and Acc. This improvement is large considering that the best classical machine learning algorithms had accuracies over 95% and that they are based on more than 20 years of expert knowledge on ECG feature engineering. Moreover, mixed solutions, obtained by either stacking classifiers or mixing handcrafted and CNN features, could yield further improvements in BAC and Acc, as shown by the preliminary experiments of Section 4.5.

One of the advantages of deep learning solutions is the capacity of the algorithms to learn discriminating features exploiting all information hidden in the ECG. This avoids the time-consuming feature extraction processes and, most importantly, improves the quality of the extracted features. The latter is well reflected by the AUCs on Table 2. Four of the ten features extracted by the deep learning architecture show a higher discrimination capacity than SampEn($d_{3}$), which is the best handcrafted feature for shock/no-shock decisions during CPR in the available literature [25,26].

Two factors were key to improve the performance of the CNN based methods from the preliminary results communicated previously in [53]. First, the design and optimization of the parameters of the CNN to obtain a better model for classification. Second, increasing the size of the database by adding 1186 new annotated samples (a 55% increase in dataset size). These led to 0.5-points and 0.3-points increases in BAC and Acc respectively, of which 0.4-points and 0.1-points are attributable to the larger dataset. And there is further room for improvement from combining the knowledge gained from deep learning and handcrafted ECG feature extraction, basic examples are shown in Table 3 which added an extra 0.5-points in Acc. The performance of deep learning solutions improves as they are exposed to more data, whereas the accuracy of classical machine learning algorithms stagnate past a given sample size. The model presented in this study overfits when more than 3 CNN blocks are used (Figure 4) since from then on the number of trainable parameters is too large for the size of the available dataset. Adding more data would help to develop deeper networks and thus to the extraction of more sophisticated features. There is therefore room to improve the deep learning models for rhythm analysis during CPR, as more and more data is recorded every day and made available in centralized repositories. In research on OHCA, the Resuscitation Outcome Consortium (ROC) network provides the largest OHCA data repository, which includes recordings of eleven regional clinical centers. However, labeled OHCA data are scarce, and obtaining quality controlled rhythm annotations from clinicians is expensive and time consuming. As an alternative, semi-supervised learning could be an efficient way to augment training data and obtain better deep learning models in the future.

As Figure 6 shows, CNN features provide more separate clusters than the handcrafted features for the shock/no-shock classes. Moreover, the deep learning model shows a quite high separability between the features corresponding to AS, OR and shockable rhythms. Therefore, in the future CNN models could improve the accuracy of classical machine learning-based multiclass rhythm classifiers. These classifiers have been demonstrated for clean [24,52] and artifacted ECGs [25], and are multilabel classification algorithms that classify the ECG into the 5 OHCA rhythm types. These algorithms are important for research to analyze large sets of OHCA data [24], and could also help clinicians during OHCA treatment as clinical support tools. The best OHCA multiclass algorithms have unweighted mean sensitivities of 78% for clean ECG [24], and of 72% if the analysis is done during CPR [25]. There is therefore margin for improvement using methods based on deep learning if sufficiently large quality controlled annotated datasets become available.

## 6. Conclusions

This paper introduces the first shock/no-shock decision algorithm during CPR based on deep learning methods. This solution improves the accuracy of the best classical machine learning models based on handcrafted features, and is able to give a shock/no-shock diagnosis compliant with AHA recommendations for shockable and nonshockable rhythms. Moreover, deep learning algorithms have room for improvement if larger annotated datasets become available allowing the design of deeper networks. This may lead to the first practical solutions for rhythm analysis during CPR, eliminating the no-flow intervals for rhythm analysis and contributing to improve OHCA survival rates.

## Figures and Tables

**Table 1:** Performance metrics for the CNN and the baseline models. The results are shown as median and 90% confidence interval (CI).

Metric	3 s			9 s	
	CNN	Baseline		CNN	Baseline
**Se**	93.2 (92.2–94.0)	93.1 (92.6–93.6)		95.8 (94.6–96.8)	95.2 (94.7–95.7)
**Sp**	94.5 (94.1–94.9)	94.1 (93.9–94.3)		96.1 (95.8–96.5)	95.6 (95.2–95.9)
AS	93.1 (92.6–93.7)	92.5 (92.2–92.8)		95.4 (94.9–96.0)	94.5 (94.1–95.0)
ORG	95.6 (95.1–96.0)	95.3 (95.1–95.6)		96.8 (96.2–97.4)	96.4 (96.0–96.8)
**BAC**	93.8 (93.4–94.3)	93.6 (93.3–93.9)		96.0 (95.5–96.5)	95.4 (95.0–95.7)
**Acc**	94.3 (94.0–94.6)	93.9 (93.7–94.1)		96.1 (95.7–96.4)	95.5 (95.2–95.8)
**PPV**	78.5 (77.2–79.6)	77.2 (76.5–77.7)		84.3 (82.8–85.6)	82.2 (81.0–83.2)
**NPV**	98.5 (98.3–98.7)	98.5 (98.3–98.6)		99.1 (98.8–99.3)	98.9 (98.8–99.1)

**Table 2:** Mean (standard deviation) of the AUCs for the CNN features and the handcrafted features obtained using 500 bootstrap replicas of the data.

CNN Features			Handcrafted Features	
Feature	AUC		Feature	AUC
$f_{6}$	97.2 (1.1)		SampEn($d_{3}$)	90.6 (2.0)
$f_{10}$	96.4 (1.6)		$\sigma (|\Delta d_{4}|)$	90.3 (1.7)
$f_{1}$	95.2 (2.6)		$\sigma (|d_{4}|)$	87.7 (1.8)
$f_{5}$	94.8 (2.3)		$\sigma (|d_{3}|)$	86.2 (2.3)
$f_{9}$	90.7 (3.7)		VFLeak	85.9 (2.7)
$f_{3}$	81.2 (11.1)		SampEn($d_{4}$)	84.8 (2.4)
$f_{8}$	75.2 (10.6)		$\bar{|\Delta d_{3}|}$	84.6 (2.8)
$f_{4}$	73.9 (8.6)		x4	82.5 (3.6)
$f_{7}$	66.9 (6.2)		$\sigma (|s_{\mathrm{den}}|)$	82.4 (2.0)
$f_{2}$	59.3 (17.1)		SampEn($d_{6}$)	80.6 (2.7)

**Table 3:** Performance metrics for 9 s segments of the mixed solutions. The results are shown as median and 90% confidence interval (CI).

Metric	CNN	Mixed Classification Solutions		
		CNN+RF	All-Features	Stacked
**Se**	95.8 (94.6–96.8)	95.3 (93.9–96.2)	95.6 (94.6–96.4)	96.1 (95.1–96.8)
**Sp**	96.1 (95.8–96.5)	96.7 (96.3–97.1)	96.8 (96.5–97.1)	96.7 (96.3–97.1)
AS	95.4 (94.9–96.0)	95.9 (95.4–96.5)	96.1 (95.6–96.6)	95.9 (95.3–96.4)
ORG	96.8 (96.2–97.4)	97.2 (96.7–97.7)	97.3 (96.9–97.7)	97.4 (96.9–97.9)
**BAC**	96.0 (95.5–96.5)	96.0 (95.3–96.5)	96.2 (95.7–96.7)	96.4 (95.9–96.8)
**Acc**	96.1 (95.7–96.4)	96.4 (96.0–96.7)	96.6 (96.3–96.9)	96.6 (96.3–96.9)
**PPV**	84.3 (82.8–85.6)	86.0 (84.6–87.4)	86.5 (85.3–87.8)	86.3 (84.8–87.5)
**NPV**	99.1 (98.8–99.3)	99.0 (98.7–99.2)	99.0 (98.8–99.2)	99.1 (98.9–99.3)

## Abbreviations

The following abbreviations are used in this manuscript:

CPR	cardiopulmonary resuscitation
CNN	convolutional neural network
RLS	recursive least squares
OHCA	out of hospital cardiac arrest
ECG	electrocardiogram
VF	ventricular fibrillation
VT	ventricular tachycardia
ORG	organized
AS	asystole
AHA	American Heart Association
CD	compression depth
TI	thoracic impedance
LMS	least mean squares
SVM	support vector machine
RF	random forest
SWT	stationary wavelet transform
TP	true positive
TN	true negative
FP	false positive
FN	false negative
Se	sensitivity
Sp	specificity
Acc	accuracy
BAC	balanced accuracy
PPV	positive predictive value
NPV	negative predictive value
SNR	signal-to-noise ratio
t-SNE	t-distributed stochastic neighbour embedding
DBi	Davies-Bouldin index
AUC	area under the receiver characteristics curve
ROC	Resuscitaion Outcome Consortium
